# Approach for Assessing the Prevalence of Psychosocial Risks of Workers in the Greenhouse Construction Industry in South-Eastern Spain

**DOI:** 10.3390/ijerph18094753

**Published:** 2021-04-29

**Authors:** José Pérez-Alonso, Marta Gómez-Galán, Marta Agüera-Puntas, Julián Sánchez-Hermosilla, Ángel-Jesús Callejón-Ferre

**Affiliations:** 1 Research Center CIMEDES, Agrifood Campus of International Excellence (CeiA3), Department of Engineering, University of Almería, 04120 Almería, Spain; mgg492@ual.es (M.G.-G.); martaaguerap@gmail.com (M.A.-P.); jusanche@ual.es (J.S.-H.); acallejo@ual.es (Á.-J.C.-F.); 2 Laboratory-Observatory for Andalusian Working Conditions in the Agricultural Sector (LASA), Avda. Albert Einstein, 4. Isla de la Cartuja, 41092 Seville, Spain

**Keywords:** psychosocial risks, occupational health and safety, health risks, construction, greenhouses

## Abstract

This paper shows the prevalence of psychosocial risks for workers in the greenhouse construction industry in south-eastern Spain. *Method*: The assessment of the workers’ psychosocial risks was carried out through simple random sampling, which uses a questionnaire containing 13 variables characterizing the companies, 14 variables characterizing the workers, and 15 questions proposed by the Mini Psychosocial Factors (MPFs) risk assessment method. A descriptive analysis and multiple correspondence analysis were performed on the sample data. *Results*: Greenhouse construction businesses in south-eastern Spain can generally be classified as small companies with an average annual turnover below EUR 2.0 million (69.3%), an average of 22.8 workers with an average age of 39.84 years old, most of whom are married, with an average of 1.76 children. The prevalence of workers at high risk was 2.9%, while 45.1% were at medium-high risk. Of the 12 psychosocial factors assessed using the MPF method, 7 of them presented a high level of worker risk: Mobbing (3.2%), Relationships (1.6%), Recognition (1.6%), Autonomy (12.9%), Emotional (8.0%), Control (4.8%), and Demands (3.2%). Lastly, the variables were grouped into four clusters, showing that larger companies are correlated with a medium (workers over 40 years of age or less than 25 years of age) to high (workers under 25 years of age) risk level in several of the psychosocial factors assessed for workers who are Spanish nationals, while in smaller companies, the workers are usually middle aged (between 25 and 40 years old) and from Eastern Europe or Africa, presenting either a low or high level of risk depending on the psychosocial factors and tasks performed. *Impact of the results*: The study reveals a lack of prevention management regarding psychosocial risks. Therefore, it is necessary to carry out new prevention programmes that optimise the psychosocial conditions of the workers, involving the workers, employers, and other social agents.

## 1. Introduction

### 1.1. Occupational Safety in Greenhouse Construction Companies in the Fruit and Vegetable Production Sector of Almería (South-Eastern Spain)

The main socioeconomic sector in the Province of Almería (south-eastern Spain) is agriculture, based on the greenhouse cultivation of fruit and vegetable products under plastic. Greenhouses are agricultural constructions that facilitate the microclimatic conditions that the crops need. According to the analysis on the fruit and vegetable campaign for Almería [1], the total production in the 2017/18 season was 3,256,594 tonnes, with a value of more than EUR 1.924 billion, of which 80.1% of the produce was exported. The total area currently under greenhouse production in the province of Almería is 32,084 ha. [2], with the main types of greenhouses being “*Almería plano*” and “*Almería raspa y amagado*” (94.3%) and, to a lesser extent, industrial greenhouses, the so-called multi-tunnel type [3]. The greenhouses are nearly 100% dedicated to the cultivation of tomato, pepper, melon, watermelon, eggplant, zucchini (courgette), cucumber, and beans [4]. Therefore, the greenhouse construction subsector in Almería is essential since it builds and maintains the main infrastructure on which this production system is based. However, these low-cost structural systems can cause certain environmental problems and especially occupational accidents [5,6,7], the same as in the general construction sector [8,9].

Worldwide, the construction sector sustains a high level of workplace accidents [10,11,12,13,14,15,16,17,18,19,20,21,22,23,24]. In June 2017, the Spanish construction sector presented a total incidence rate of 7347.0, followed by the industrial sector (5240.0), the agricultural sector (5117.0), and the service sector (2520.0) [25]. As an integral part of the construction sector, the greenhouse construction subsector likewise endures a high workplace accident rate. For this reason, Pérez-Alonso et al. [7] studied the incidence rate in the Almerian greenhouse construction sector and compared it to that in the Almerian construction sector for the period 1999–2007- for all years, the rate was higher in the greenhouse construction sector than in the construction sector, except in 2002 and 2005. Over this period, a decreasing trend was observed in the greenhouse construction incidence rate but with fluctuations. There are currently no up-to-date incidence rate data for the greenhouse construction subsector because accident data are not disaggregated into subsectors. Pérez-Alonso et al. [26] also studied the overall potential risks faced by workers in greenhouse construction in terms of the workplace, concluding that they presented a significant or moderate rating for risks involving falling from different levels, objects falling from the same or a different level, being knocked down, or other accidents with vehicles. However, in-depth studies looking specifically at other risks were advised—risks that were, in principle, considered tolerable in an overall assessment, such as psychosocial risks, heat stress, and noise risk. Even though Pérez-Alonso et al. [5,27] carried out a risk assessment in the greenhouse construction subsector for the last two of these risks, respectively, none have yet been carried out for psychosocial risks. Due to this lack of knowledge regarding the condition of psychosocial risks for greenhouse construction workers in south-eastern Spain, a study is required to determine the prevalence of these risks in the workers and to identify the relationships between these psychosocial factors, as well as the sociodemographic variables of the workers and those that characterise the companies. This would allow recommendations to be made for preventing the appearance of such risks if they are incorporated into the company’s preventive management.

### 1.2. Psychosocial Risks

In recent decades, there have been significant changes in the world of work, closely linked to labour organisation and management [28], which have resulted in emerging risks in the area of occupational safety and health, such as psychosocial risks [29,30,31]. The effect of the psychosocial environment at work on physical and mental health has been well documented [28,32,33,34,35,36,37,38,39,40,41,42]. Linked to psychosocial risks, issues such as work-related stress are widely recognised as important challenges for occupational health and safety [31]. As indicated by Leka et al. [28], psychosocial risks are defined by the International Labour Organisation in terms of interactions between the content of the position, the work organisation and management, and other environmental and organisational conditions, on the one hand, and the competencies and needs of employees on the other. Psychosocial risks are associated with experiencing work stress, which refers to the response people may have when they are presented with work demands and pressures that do not correspond to their knowledge and skills, and which challenge their capacity to cope [43].

Psychosocial risks can lead to emotional reactions (irritability, emotional withdrawal, anxiety, sleep problems, depression, burnout), cognitive reactions (difficulty in concentrating, remembering, making decisions, decreased creativity), behavioural reactions (abuse of drugs, alcohol, and tobacco; destructive behaviour, loss of motivation), and are also associated with physiological reactions, such as musculoskeletal disorders, particularly back problems, weakened immunity, peptic ulcers, heart problems, or hypertension [40]. Protracted exposure to stress can therefore have serious negative consequences for the individual and lead to a loss of well-being for them as well as members of their household. The performance of organisations is likely to worsen. Society suffers from a loss of capacity for work, direct costs linked to health spending, and loss of quality of life [40]. In this context of psychosocial risks, the study of harassment and violence at work deserves particular attention; for this, the European Union developed an autonomous framework agreement [42].

As stated by Llaneza-Álvarez [44], psychosocial risk factors do exist; they are not figments of the worker’s imagination but are part of the working conditions and must be in the initial/integral risk assessment. Thus, a psychosocial factor understood as ‘*conditions that lead to stress at work and other problems that have an impact on health*’ could be said to be a multicausal factor since it includes aspects relating to the job and the work environment, to the cultural or organisational climate, to work functions, to interpersonal relationships, to the design and content of tasks, the environment of the organisation, and aspects particular to the individual [45].

In some countries, such as New Zealand [46], Germany [47], and Belgium and France [48], the legislation explicitly mentions mental health problems as well as explicit regulatory provisions governing the prevention of psychosocial risks. European Union Council Directive 39/391/EEC on mental health in the workplace provides for this, and an interpretative document to implement that directive was developed, which also references other directives that supplement it [41]. The psychosocial assessment should, in principle, be a company requirement, regardless of their activity; for this reason, in Spain, one of the preventive principles stipulated in Article 15 (l) (d) and (g) of the Occupational Risk Prevention Act [49] justifies this and is based on it, establishing psychosocial assessment as a preventive guideline in all cases.

On the other hand, total independence does not exist between the psychosocial risks and the physical demands on the worker [50]. For instance, muscle disorder and fatigue can be a consequence of stress and the pace of work [51,52,53]. In different economic activities, the relationship between the psychosocial risks and the musculoskeletal disorders (MSDs) suffered by the workers is evident and has been scientifically proven in various sectors, such as with construction workers [54,55,56], flight attendants [57], computer scientists [58], cooks [59], health workers [60,61], and agricultural workers [62,63,64,65].

Psychosocial risk assessment is a complex process, which requires resorting to different sources of information and using different techniques. The specific methods for the psychosocial risk assessment, according to Montoya-García et al. [65] can be grouped into two types: (a) those that allow determining the state of a worker by measuring the changes in his physiological parameters (such as eye movements or cardiac variability) associated with cognitive demands of the task they perform, and (b) contrasted methods usually based on the use of questionnaires that have been contrasted in one or more sectors of production. The methods of this second group are the most applied, and specifically in Spain, the following have been used and contrasted mainly according to Montoya-García et al. [65], where information about them can be expanded: Mini Psychosocial Factor (MPF) [66], FPSICO [67], ISTAS21 [68], FP-ISR [69], PSICOMAP [70] and RED-WONT [71].

Finally, in relation to psychosocial factors affecting construction workers, there are many studies that address this issue. For instance, psychosocial risk exposure has been studied by various authors [72] in different work sectors. Although the traditional approach to occupational health and safety focuses mainly on physical risks, psychosocial factors are presented as potential targets for the prevention of workplace injuries and diseases [73]. The risk of an accident at work in the construction sector is linked to psychosocial factors such as a hostile environment and economic insecurity at work [73,74], as well as control, harassment, and discrimination at work [75].

According to Salanova et al. [76], the main factors in the construction sector are overconfidence on the part of more experienced workers and the lack of experience of young people, as well as certain labour demands such as the routine and being overworked; these factors can be both qualitative and quantitative. In addition, mobbing, leadership, and role conflict are the most influential factors in workers’ health [77,78] and on their productivity [79]. In this sense, overly exigent labour demands, lack of control over the work, and poor social support are also factors related to productivity [80]. Stress is likewise related to productivity, and most stress-dealing behaviours were of the emotional type in construction workers [81]. There is also a link between work tension and illness-induced absenteeism [82]; thus, several authors have linked psychosocial risks to absenteeism in the construction sector, with the climate of insecurity, harassment [75], and leadership and exhaustion [77] being determined as the most influential factors. Age, obesity, smoking, and lack of control at work [56], as well as economic factors [83], are also decisive when it comes to being absent from work.

The age and experience of workers in the workplace also play an important role in assessing risks. According to Hoonakker and Van Duivenbooden [84], and Maqsoom et al. [79], younger workers are more susceptible to psychosocial risks and labour demands in the construction sector. Similarly, in Australia, it was concluded that psychosocial factors at work can determine the psychological health of younger workers [85], who are also more prone to drug use. On the other hand, greater work experience in the sector is related to higher levels of job satisfaction [86].

## 2. Objectives

The aim of this paper is to determine the prevalence of psychosocial risks in workers in the greenhouse construction industry in south-eastern Spain (Almería), as well as the relationship between the workers’ sociodemographic variables, the company characteristics, and the different psychosocial factors that affect them. Finally, the aim is to propose recommendations that prevent these risks from occurring.

## 3. Materials and Methods

### 3.1. Research Design and Data Analysis

To assess the prevalence of psychosocial risks in workers in the greenhouse construction industry in south-eastern Spain (Almería), a questionnaire consisting of two distinct parts was prepared. The first consists of 13 variables characterizing the greenhouse construction companies in Almería (for employers), as a prior step to assessing the psychosocial risks of their workers, while the second part consists of 14 variables concerning the personal characteristics of the workers in these companies and 12 psychosocial factors that are evaluated using the Mini Psychosocial Factor (MPF) method [66] based on the 15 questions proposed by this method (for workers). The description of the Mini Psychosocial Factor Method is shown in Section 3.3.2. The prepared questionnaire is shown in Appendix B.

The inclusion criteria for the workers in the study were that they had reached the age of consent, were legally employed by the company, and had been working at the greenhouse construction company for at least a year. Workers would be excluded if they failed to meet any of the three criteria.

Field data acquisition was carried out by the simple random sampling of the companies and workers in the study using the questionnaire designed for this purpose, and all the information was subsequently converted into a database format. With the data corresponding to the 15 MPF-method questions, the assessment results for workers’ psychosocial risks were obtained through a computer application available for this purpose, ceded by the authors [66].

Additionally, a descriptive analysis of the sample data was carried out using the SPSS v.23 (IBM, New York, NY, USA) programme on all the variables characterising greenhouse construction companies and workers, as well as the values obtained from the MPF assessment of the 12 psychosocial factors analysed. A variance analysis was also carried out on the variables characterising the companies along with a multiple correspondence analysis of the most significant company and worker characteristic variables, and those for the worker’s psychosocial assessment using MPF, thus obtaining the relationships that exist between them.

Prior to the above analysis, it was confirmed that the sample data verified the independence, homoscedasticity, and normality conditions of the variables.

Finally, regarding the study’s ethics statement, all subjects gave their informed consent before they participated in the study. The study was conducted in accordance with the Declaration of Helsinki, and the protocol was approved by the Bioethics Committee of the University of Almería (UALBIO2021003).

### 3.2. Sampling of Almerian Greenhouse Construction Workers and Companies

#### 3.2.1. Census of Greenhouse Construction Companies in Almería

To prepare a census of greenhouse construction companies, the corresponding information was requested from the Almería Chamber of Commerce, Industry, and Navigation. The Cajamar report “Analysis of the fruit and vegetable campaign in Almería, 2017/18” [1] was also consulted; this shows the number of greenhouse construction companies in Almería. Furthermore, according to the National Classification of Economic Activities (CNAE) called CNAE-2009 [87], the construction sector is included in Section F; however, greenhouse construction companies are inscribed in different sections depending on whether they also carry out other activities besides greenhouse construction such as irrigation installation, agricultural machinery sales, and the construction of warehouses.

The census obtained includes 30 companies located in the following municipalities of Almería Province: El Ejido (33.33%), Adra (3.33%), Berja (6.67%), Vicar (10.00%), La Mojonera (6.67%), Roquetas de Mar (6.67%), Alhama de Almería (6.67%), Pulpí (3.33%), Nijar (13.33%), and Almería (10.00%).

#### 3.2.2. Census of Greenhouse Construction Workers in Almería

To determine the number of workers employed in Almerian greenhouse construction companies, a request was made to the companies themselves as well as to the Social Security and Treasury Delegation in Almería; however, the latter entity does not have data on greenhouse construction workers that are disaggregated from other construction workers. From the company information obtained, the number of greenhouse construction workers registered in Almería was 684, an average of 22.8 workers per company.

#### 3.2.3. Sample Size

The sample size was calculated to determine the proportion of greenhouse construction workers presenting a high psychosocial risk level, considering the average prevalence of high-level risks in each of the 12 psychosocial factors analysed by the MPF assessment method. On the other hand, this same high-level prevalence average, which is 17.96% (the average calculation of the 12 high-risk values for the 12 psychosocial factors studied), is known for workers who work in Almerian greenhouse crop cultivation [65]; furthermore, immigrants make up a high percentage of the workers in this sector, as they do in the greenhouse construction sector.

Therefore, taking 684 as the registered number of greenhouse construction workers in Almería, an accuracy of 5.0%, a confidence level of 95%, and an expected frequency of 17.96% as the factor to be estimated, the sample size was initially set at 170 workers. However, this number was impossible to achieve because of circumstances regarding the concentration of work during the months that the survey was conducted. In the end, only 62 workers were interviewed, setting the final accuracy at 5.72%, by virtue of the prevalence determined.

#### 3.2.4. Sampling Plan

The sampling phase was carried out from 3 June to 30 August 2019 using simple random sampling, surveying the managers and workers in the Almerian greenhouse construction companies. For this purpose, the company headquarters were visited as well as the agricultural sites where the greenhouses were being built. To set the day of the visit, the researchers contacted the company managers by phone and/or email and agreed on the day and time of the visit with their workers.

To validate the functioning of the developed questionnaire, three employers and three workers completed it prior to the sampling itself; this was carried out to see if any format changes were required. From this, minor deficiencies were observed and rectified, resulting in the questionnaire shown in Appendix B.

### 3.3. The Psychosocial Risk Assessment Method Used

#### 3.3.1. Selecting the Psychosocial Assessment Method

To choose the assessment method to use in this work, a decision matrix (Table A1, see Appendix A) was constructed that assessed the various aspects of the selected methods, starting from the decision matrix described by Montoya-García et al. [65], which proposed the following assessment methods: FPSICO v.1. [67], ISTAS21 [68], Mini Psychosocial Factor (MPF) [66], FP-ISR [69], PSICOMAP [70], and RED-WONT [71].

Specifically, in this work, the decision matrix weighting was scored between 1 and 4. The parameters considered for the weighting were: the speed of completing the questionnaire by the workers, the number of questionnaire questions, its applicability to the construction sector, and the reliability and ease of statistical handling. The assessment methods adopted for the weighting were the six contrasted psychosocial methods described above by Montoya-García et al. [65], which are the most used in Spain.

The results obtained in Table A1 (see Appendix A) justified the choice of the MPF method, followed by FPSICO (v.4) and RED-WONT. Of the three, MPF was chosen. In addition to it obtaining a better score when all three methods were tested on four greenhouse construction workers, it was the MPF method that proved most operational, both for its greater speed and for being better understood by the workers; these aspects are especially important since, in most cases, the questionnaire has to be filled out while the workers are on-site performing their greenhouse construction work.

#### 3.3.2. Description of the Mini Psychosocial Factor Method (MPF)

The MPF method [66] assesses 12 variables (Rhythm, Mobbing, Relationships, Health, Recognition, Autonomy, Emotional, Support, Compensation, Control, Demands, and Mental Load), by answering the 15 questions proposed in the questionnaire. A description of the 15 questions and the 12 variables can be found in Ruiz and Idoate [66] and Montoya-García et al. [65], as well as the scales used to determine the variable scores from the responses given to the 15 questions (the scale shown in Table 1). Montoya-García et al. [65] present some examples in which the MPF method has been used and validated in Spain: the Higher Council for Scientific Research in Spain (CSIC), the University of Almería, ArcelorMittal Spain, the Navarro Health Service, and the Railway Infrastructure Administration (ADIF).

## 4. Results

### 4.1. Descriptive Analysis of the Variables

#### 4.1.1. Characterisation of Greenhouse Construction Companies

Table A2 (see Appendix A) shows the frequencies for each of the variable categories characterising Almería’s greenhouse construction companies.

Table A3 (see Appendix A) shows the construction tasks for which specialised worker crews can be formed, with the nomenclature code used to reference them in Table A2 (see Appendix A), which shows the most common possible crew combinations formed by the companies.

#### 4.1.2. Characterisation of the Workers in Greenhouse Construction Companies

Table 2 shows the frequencies for each of the various categories that characterise workers in Almería’s greenhouse construction companies. In addition, for the workers’ sociodemographic quantitative variables, their mean and standard deviations are shown.

#### 4.1.3. Characterisation of the Psychosocial Risk Prevalence for Workers in Greenhouse Construction Companies

Table 3 shows the results of the descriptive analysis performed on the scores obtained for the 12 psychosocial factors that were assessed using the MPF method. These scores came from the method’s 15-question questionnaire provided to the Almerian greenhouse construction workers. Table 3 also shows the risk rating performed for the average value obtained in each factor: High (H), Medium (M), or Low (L) risk. However, it is not these average values that are significant in interpreting the psychosocial assessment but rather the frequencies showing the percentage of workers presenting a certain level of risk (High (H), Medium (M), or Low (L)) for each of the 12 psychosocial factors assessed, as shown in Table 4.

### 4.2. Multiple Correspondence Analysis

This section sets out the results of the multiple correspondence analysis carried out on the most significant variables of the workers’ characteristics and the general company variables, together with the 12 psychosocial factors assessed by the MPF method. Table A4 (see Appendix A) shows the nomenclature for the categories of the general company variables and worker variables, while Table A5 (see Appendix A) shows those pertaining to the 12 psychosocial factors.

The results from the multiple correspondence analysis, performed for the representative variables shown in Table A4 and Table A5 (see Appendix A), allows us to identify the correlations between the variable categories, as well as between the variables themselves, by means of a two-dimensional model summarising the information of all the variables analysed within it.

The model that was obtained from this analysis has two significant dimensions—The first explains 29.502% of the variance with a Cronbach α coefficient of 0.886 and an eigenvalue of 6.490, and the second dimension explains 18.846% of the variance with a Cronbach α coefficient of 0.795 and an eigenvalue of 4.146; therefore, for the factorial model as a whole, the mean of the variance explained is 24.174%, with an average Cronbach’s α coefficient of 0.851 and an average eigenvalue of 5.318, signifying that the model’s reliability is good.

Table 5 shows each variable’s discrimination measurements for each of the two dimensions, along with the average. Each discrimination measurement coincides with the variance in each dimension’s coordinates for the modalities of each variable so that those variables whose modalities have coordinates on a different dimension from each other present high measures of discrimination in that dimension. Likewise, a variable with similar discrimination measurements in the two dimensions reflects the difficulties in assigning it to a given dimension [6,22,23].

**Figure 1:**
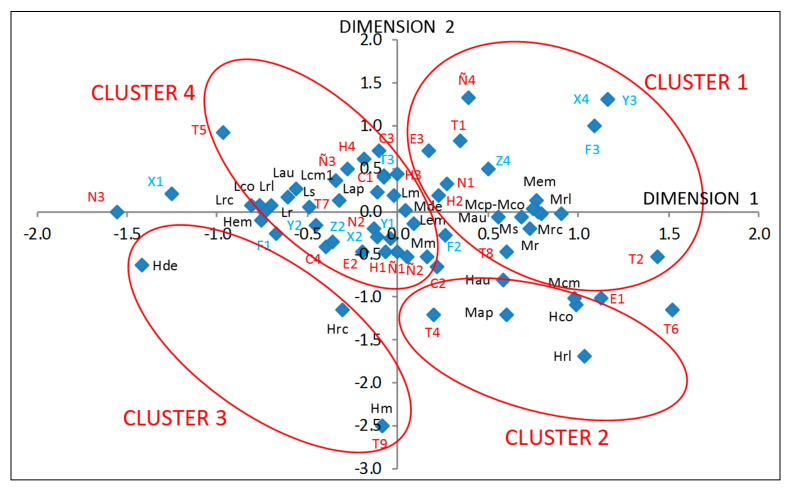
Factorial plane of the variable category relationships studied in the multiple correspondence analysis (see nomenclature in Table A4 and Table A5 (Appendix A)).

The multiple correspondence analysis carried out allows us to identify the categories of each variable that best discriminates the objects (the workers); thus, the quantifications of the variables are obtained and represented in a factorial plane, in which the axes are the two dimensions obtained in the model (Figure 1). Category quantifications are the average of the scores for objects in the same category. By using the factorial plane representation (Figure 1), we can observe the correlations or correspondences of the variable categories.

## 5. Discussion

This epigraph discusses the results achieved, showing that the set objectives have been met and the preventive recommendations have been proposed. To this end, the discussion is structured under the following headings:

### 5.1. Characterisation of Greenhouse Construction Companies

As shown in Table A2 (see Appendix A), the corporate structure for 100% of the sampled enterprises is that of the limited company. Of these, 46.1% of the sampled companies only construct Almería Type greenhouses, 15.4% construct both Almería type and multi-tunnel (industrial-type) greenhouses, and finally, 38.5% construct all kinds of greenhouses as well as carrying out other activities typical of the auxiliary agricultural industry, such as irrigation installation, air conditioning, and building warehouses. The average number of years that the companies have been constructing greenhouses is 21.8 (±9.6) years, and as can be seen in Table A2 (see Appendix A), 38.4% have been in operation for between 10 and 19 years, 30.8% for between 20 and 30 years, 23.1% of them for over 30 years, and 7.7% for less than 10 years. In addition, the average number of workers in the sampled companies is 22.8 (±27.3), a much higher value than in 2008, when it was 13.3 [6]. As Table A2 (see Appendix A) shows, 61.5% have between 10 and 19 workers, 7.7% have between 20 and 50 workers, and it shows the same percentage (15.4%) both for those with more than 50 workers and for those with fewer than 10 workers. Comparing these data with those obtained by Pérez-Alonso et al. [6] in 2008, companies have now migrated largely from the group with less than 10 workers to the group with between 10 and 19, given that, in 2008, it was 50% in the first group and 20% in the second.

The average number of immigrant workers in the sampled companies is 16.0 (±20.3) and, as can be seen in Table A2 (see Appendix A), 46.1% of companies have between 10 and 19 immigrants, and 38.5% of companies have fewer than 10 immigrant workers. Of these, 46.1% of the companies employ only Romanian immigrants, 7.7% employ Romanians and sub-Saharan Africans, 38.5% employ Romanians, Russians, and North Africans, and finally, 7.7% employ Romanians, Russians, North Africans, and Latin Americans. The data on immigrant workers accord with those reported by Salanova et al. [76] for the construction sector in Spain in 2006, in which 10% of the total immigrant workers worked in the construction sector.

The average number of office workers in the sampled companies is 1.8 (±2.3), and as shown in Table A2 (see Appendix A), 69.2% have between one and five office workers, 23.1% do not have any, indicating that they contract management out to external management companies, and 7.7% have between 6 and 10 office workers. In contrast, the average number of workers of the sampled companies who work on-site or in the field is 21.1 (±25.9), and as can be inferred from Table A2 (see Appendix A), 61.5% employ between 10 and 19 site workers, 23.1% employ less than 10, and 15.4% employ more than 50 site workers.

As to whether companies have crews of operators specialised in each of the tasks carried out in the greenhouse construction procedures, Table A2 (see Appendix A) shows that 100.0% of them do.

As can be observed in Table A2 (see Appendix A), there are four combinations of crew configuration that are presented at 15.4% each. These combinations are 3 + 4 + 5 + 8 + 11, 3 + 4 + 5 + 6 + 8 + 9 + 11, 3 + 4 + 5 + 6 + 8 + 9 + 10 + 11, and 2 + 3 + 4 + 5 + 6 + 7 + 8 + 9 + 11 (see Table A3). In addition, 23.0% of companies form crews that combine all the construction tasks (2 + 3 + 4 + 5 + 6 + 7 + 8 + 9 + 10 + 11) (see Table A3). It should be noted that only 7.7% of companies form fewer than five crews, compared to 40.0% in 2008 [6].

With regard to the working period of the companies throughout the year, Table A2 (see Appendix A) shows that 100.0% construct or maintain greenhouses every month of the year. This contrasts with the data from 2008 when the companies only worked for an average of 6.5 months per year [6]. Additionally, as for annual turnover, 46.2% of the companies have an annual turnover of between EUR 0.5 and 1.0 million, 23.0% of between EUR 1.0 and 2.0 million, 7.7% of between EUR 3.0 and 5.0 million, and finally, 23.1% have a turnover of over EUR 5.0 million. Therefore, companies that turnover less than EUR 2 million a year represent 69.3% of the total, practically the same percentage as these companies turned over in 2008, which was 70.0% [6]. However, the remaining 30.7% of companies turned over more than in 2008. Given the distribution of turnover and the total number of workers that the companies have, 15.4% of Almería’s greenhouse construction companies can be considered as microenterprises, another 15.4% as medium-sized enterprises, and the remaining 69.2% as small enterprises.

Finally, Table A2 (see Appendix A) indicates that 38.5% of the companies are assigned to code 4391 (roof construction) of the CNAE-2009 classification [87], 53.8% to 4399 (other specialised construction activities, not specified elsewhere) and finally 7.7% are assigned to code 4121 (construction of residential buildings). One can observe therefore that, of the 23 classes in the CNAE-2009 grouping [87] for construction (Section F), the greenhouse construction companies of Almería are only assigned to three of them.

### 5.2. Characterisation of the Workers in Greenhouse Construction Companies

As can be inferred from Table 2, 3.2% of the sampled workers are under the age of 25 years old, 54.9% are between 25 and 40 years old, and 41.9% are over 40 years old. The average age of the workers is 39.84 (±9.70) years old, which is higher than that reported by Salanova et al. [76] in a sample of construction workers in the community of Valencia (Spain), which was 31 years old. The mode for the average age of greenhouse construction workers in Almería is between 25 and 40 years old, the same as for workers who work in greenhouse crop cultivation in Almería [65]. In addition, 100.0% of the workers are men. This coincides with what happens in the building and civil engineering sectors throughout Spain [76]. In contrast, for workers who carry out greenhouse crop cultivation in Almería, 29.35% of them are women [65].

With regard to the workers’ weight, Table 2 indicates that 30.6% weigh between 70 and 80 kg, 29.0% between 80 and 90 kg, 22.6% between 90 and 100 kg, 6.5% weigh more than 100 kg, and 11.3% weigh less than 70 kg, the mode being the group between 70 and 80 kg. The average weight of workers is 83.16 (±11.50) kg. Additionally, in terms of height, 53.2% are between 1.7 and 1.8 m tall, 37.1% between 1.8 and 1.9 m tall, and finally 9.7% are less than 1.7 m tall. The mode is in the range between 1.7 and 1.8 m tall, and the average height of the workers is 1.76 (±0.06) m.

As regards the marital status of the workers, Table 2 indicates that 53.2% of the sampled workers are married, 33.9% are single, 4.8% are divorced and 8.1% have a common-law partner. The mode in the marital status of the workers is to be single. In addition, 43.5% of the workers have no children, 24.2% have one child, 19.4% have two children, and the remaining 12.9% have more than two children. The mode in the number of children that workers have is to have none, and the average is 1.06 (±1.18).

As regards the workers’ nationality, Table 2 shows that 59.7% of workers are from Eastern Europe, predominantly Romanians, 38.7% are Spanish, and the remaining 1.6% are Africans. As is clear from these results, greenhouse construction in Almería is dominated by workers from Eastern Europe (especially Romanians, followed by Russians), unlike workers who work in greenhouse crop cultivation in Almería, where they are predominantly African [65]. This contrasts with the sample of construction workers from the community of Valencia (Spain), where 82% of workers are Spanish nationals [76]; thus, the greenhouse construction sector has a higher percentage of foreign workers.

Regarding the workers’ specialisation, in terms of the tasks carried out in greenhouse construction, Table 2 (and the nomenclature in Table A3, see Appendix A) shows that 43.5% of the sampled workers work in 5 + 6 crews (installation of wire elements + placement of storm guttering), 14.5% in 3 + 4 + 5 + 6 crews (masonry + foundations and casting + installation of wire elements + placement of guttering), 9.7% in 4 + 5 + 6 + 7 + 9 crews (foundations and casting + installation of wire elements + placement of guttering + placement and changing plastic + placement of windows), 8.1% in the 7 crew (placement and changing plastic), another 8.1% in 3 + 4 + 5 + 6 + 9 + 10 + 11 crews (masonry + foundations and casting + installation of wire elements + placement of guttering + placement of windows + assembly of multi-tunnel greenhouses + transport of materials and operators), 6.5% in the 8 crew (welding on metal elements), 4.8% in 4 + 11 crews (foundations and casting + transport of materials and operators), 3.2% in the 2 crew (hole drilling) and the remaining 1.6% in the 9 crew (window placement).

Moreover, as regards accidents suffered by workers over the past year in the greenhouse construction industry, Table 2 shows that 85.5% of sampled workers indicate that they have not experienced any accidents at work in the last year. However, 4.8% say that they suffered blows and falls over the same period, another 4.8% suffered falls at the same level, 1.6% suffered falls from height, and 3.3% suffered burns from coming into contact with a thermal agent. In terms of accidents suffered outside the workplace, 93.6% of workers indicated that they have not suffered any; however, 3.2% say they have tripped, 1.6% have fallen from the same level, and the remaining 1.6% have been in road accidents. It should be noted that there are studies that link psychosocial risks to the risk of accidents at work. The factors most associated with the occurrence of construction accidents are a hostile work environment and economic insecurity at work [73,74], with control, harassment, and discrimination also being influential factors [75]. The study conducted by Goldenhar et al. [88] concludes that there are 12 factors directly related to the occurrence of accidents in construction, namely, demands, control, the security climate, economic safety, compliance with safety standards, responsibility for the safety of others, hours of work and the amount of work, as well as experience and sub-utilisation of skills.

With regard to the period off work due to accidents in the last year, Table 2 shows that, in the workplace, 95.2% of workers indicated that they had not been off work, while 3.2% indicated that they were off work for between 8 and 30 days, and 1.6% were off work for between 1 and 7 days. For accidents occurring away from the workplace, 95.2% of workers indicated that they had not been off work in the last year, while 3.2% indicated that they were off work for between 1 and 7 days, and 1.6% were off work for between 8 and 30 days. As noted, periods off work caused by work accidents in greenhouse construction workers are small and also appear in a small percentage of workers; however, in the construction sector in general, there are studies showing that these workers can even enjoy a disability pension; for instance, two studies [89,90] found that although the main reasons for the pension are musculoskeletal disorders, psychosocial conditions at work can cause illnesses resulting in the granting of disability pay.

With regard to workers’ illnesses originating from their work in greenhouse construction, Table 2 shows that 90.3% of workers indicated that they had not suffered any illness, while 1.6% reported having herniated discs, and 8.1% suffered from low back pain; the latter value is almost the same as the 8.89% of workers affected in the lumbar region and abdomen, which was already determined in 2012 in this same sector by Pérez-Alonso et al. [7]. Therefore, from these results and those for the prevalence of psychosocial risks in greenhouse construction workers, the relationship between psychosocial and musculoskeletal risks is observed, coinciding with studies carried out in the United States by Sobeih et al. [55], and in Sweden by Engholm and Holmstrom [54], which conclude that exposure to psychosocial risks at work positively influences the development of musculoskeletal disorders. Psychosocial risks in construction workers are also linked to other diseases—Cawley et al. [91] determined that work-related stress and the family situation are linked to diastolic blood pressure and physical pain. In this regard, Hammer et al. [92] concluded that reducing exposure to psychosocial factors in construction can improve the workers’ blood pressure and thereby improve their health.

Finally, in terms of the years that workers have worked in greenhouse construction, Table 2 shows that 48.4% have been working for between 5 and 14 years, 27.4% for between 15 and 24 years, 12.9% for more than 25 years, and 11.3% for less than 5 years. The mode in the number of years working in greenhouse construction is in the range between 5 and 14 years, and the average is 13.27 (±8.73). Therefore, 59.7% of workers have less than 15 years of work experience, while 40.3% have more than 15. In this regard, a study by Pidd et al. [85] on Australian construction apprentices shows that psychosocial factors at work can determine the psychological health of younger workers, and in addition, this group is more prone to drug use. Moreover, greater work experience in the sector is related to higher levels of job satisfaction according to the study carried out by Navarro-Abal et al. [86].

### 5.3. Characterisation of the Psychosocial Risk Prevalence for Workers in Greenhouse Construction Companies

As shown in Table 4, the average percentage of workers at high risk from all 12 factors is 2.9%, while 42.2% are at medium risk, and the rest are at low risk. Therefore, 45.1% of this worker average have a medium-high risk level that requires some form of intervention. This value is much lower than that obtained by Montoya-García [65] for workers carrying out greenhouse crop cultivation in the Province of Almería, for whom the average percentage was 83.36%. For these workers, the high-risk level was always greater for the 12 psychosocial factors analysed than for the greenhouse construction workers.

By factor, Autonomy stands out, with a high level of risk for 12.9% of the workers. This highlights a lack of capacity in managing their work demands. In this regard, Canivet et al. [93] determined that, for the Swedish workforce, low decision-taking freedom, together with high psychological demands at work and labour pressure, are the most decisive factors influencing the receipt of a disability pension.

The next psychosocial factor with the highest percentage of workers at high risk is the Emotional factor, at 8.0%; however, the percentage at low risk is 46.8%, from which one can deduce a degree of emotional involvement when the workers are involved in their company’s work group, performing their daily construction tasks.

There are five psychosocial factors in which workers present a zero percentage at the high-risk level and yet the highest percentage at the low-risk level; these are Rhythm, Health, Support, Compensation, and Mental Load. This reflects that these workers consider themselves as having an acceptable state of health, that the work pace they are subjected to is tolerable, no doubt due to the work of the group or team, which provides adequate support and regards them as a person besides a worker. In addition, the intellectual requirements and effort that workers must expend to meet the work demands (the workload) appears to be below that of construction workers as a whole, whose problems with qualitative mental overload and work routine are recognised [76]. Nonetheless, 96.8% of greenhouse construction workers have a medium level of Demands risk, and the rest (3.2%) have a high level of risk, with no low-risk workers.

Finally, no appreciable Mobbing issue was observed in Almerian greenhouse construction workers, with 83.3% of them presenting a low level of risk, and only 12.9% and 3.2% presenting a medium and high level of risk, respectively. This is consistent with the results discussed for Rhythm, Health, Support, Compensation, and Mental Load since the absence, or scarcity, of Mobbing improves health [94,95] and worker productivity [79]. Productivity is also related to Social Support and Control of the work [80].

### 5.4. Relationships between the Personal Characteristics and the Level of the Workers’ Psychosocial Risk with the General Characteristics of Greenhouse Construction Companies

As can be seen in Table 5, the leading variable in the *ranking* of explanatory variables is “A” (0.6427), as it presents the highest discrimination, followed in descending order of explanation by the variables Company Turnover (F) (0.372), Mental Load (CM) (0.348), Number of Company Workers (X) (0.348) and Relationships (Rl) (0.342). The least explanatory variable is Demands (De) (0.040), followed by Nationality (N) (0.072). Regarding discrimination in both dimensions, the first dimension presents larger discriminations with the variables Recognition (Rc) (0.639), Relationships (Rl) (0.636), Control (Co) (0.595), Compensation (Cp) (0.573), Company Turnover (F) (0.482) and Rhythm (R) (0.446), while the second dimension presents large discrimination but lower than those in Dimension 1, for variables Worker Activity (A) (0.482), Number of Years Worked (Ñ) (0.457), Worker’s Age (E) (0.362), and Mental Load (CM) (0.359).

As noted above, ideally a variable has a high value in only one dimension, and a low value in another, as is the case with the variables Rhythm (R), Relationships (Rl), Health (S), Recognition (Rc), Emotional (Em), Compensation (Cp), Control (Co) and Company Turnover (F), which are more correlated with Dimension 1, and therefore, this dimension better discriminates the categories of these variables; likewise, the variables Worker’s Age (E), Worker’s Marital Status (C), Worker Activity (A), and Number of Years Worked (Ñ) are more correlated with Dimension 2, hence this dimension better discriminates the categories of these variables.

Considering all the above, four groups (or clusters) of associations can be represented between the variable categories, as shown in Figure 1. Table 6 sets out the main characteristics of the four clusters determined by virtue of the variables studied and the level of risk for each of the 12 psychosocial factors analysed.

The results obtained, based on each of the four clusters, are discussed below.

Cluster 1 associates those variable categories that have positive values in both dimensions, and also negative values in Dimension 2; it is characterised because it associates medium-level worker risk for the psychosocial factors Rhythm (Mr), Relationships (Mrl), Health (Ms), Recognition (Mrc), Autonomy (Mau), Emotional (Mem), Compensation (Mcp), Control (Mco) and Mental Load (Mcm), as well as with larger companies with a higher turnover (F3), a greater number of workers (X4), and more immigrant workers (Y3), and that the worker’s nationality would be Spanish (N1), would be more than 40 years old (3) or less than 25 years old (E1) (although this last category is located on the border with Cluster 2, as described below) and working in the construction tasks T2 (workers who put on and change greenhouse plastic) and T8 (foundations and casting + installation of wire elements + placement of gutters + changing of plastic + placement of windows), while the companies they work for would, in addition to building all kinds of greenhouses, be engaged in other activities related to construction and the auxiliary agricultural industry (Z4). Given the multiplicity of tasks that the workers associated with this cluster would perform, they present a medium level of risk in the psychosocial factors Rhythm, Relationships, Health, Recognition, Autonomy, Emotional, Compensation, Control, and Mental Load. In addition, the cluster is associated with Spanish workers with a medium level of health (there is no sample worker with a high level of health), while if observed in Cluster 4, it is associated with a low-level health risk in Eastern European workers, and even African workers, signifying that Spanish workers present a worse level of health than foreign workers, who may be more accustomed to difficult working conditions in their home countries. These results accord with those of Montoya-García [65] for Almerian greenhouse crop cultivation workers and, as cited by Cross et al. [96], this is attributed more to the social characteristics of the workers than to the occupational risk prevention policies of the companies (between different countries).

Cluster 2 brings together those variable categories studied that have positive values in Dimension 1 and larger negative values in Dimension 2, associating a small group of psychosocial factors with a high-risk (H) (Relationships (Hrl), Control (Hco), and Autonomy (Hau)) and medium-risk (M) level (Support (Map) and Mental Load (Mcm)), the latter at the border with Cluster 1, along with the task carried out by T4 workers (foundations and casting, and transport of materials and operators). The characteristics of workers in this cluster are also associated with E1, namely, workers under the age of 25 (although this category is located on the border with Cluster 1). Therefore, these construction tasks are associated with a lack of Relationships, Autonomy, and Control in these workers, and with their youth. Firstly, this seems to be logical since the workers who carry out the foundations and casting task are usually part of small crews, meaning the relationships between the workers are unfavourable, and the worker is not able to manage the demands because they are imposed upon him nor is he able to develop new skills (control), which is the same as happens to operators who transport materials since they usually work alone. Furthermore, all this is associated with younger workers, who suffer high-risk levels in the Relationships, Autonomy, and Control factors, and to a lesser extent, in Mental Load and Support, the latter two being medium-risk levels (since there is no high-level affection in any sample worker for these two psychosocial factors). This coincides with that indicated by Hoonakker and van Duivenbooden [84] and Maqsoom et al. [79], who state that the younger the worker is, the greater the affection to psychosocial risks in the construction sector. Moreover, a medium-high risk level in Support can affect productivity [80].

Cluster 3 brings together those variable categories studied that have negative values in both dimensions, associating a small group of psychosocial factors with a high-risk level (H): Demands (Hde), Emotional (Hem), Recognition (Hrc), and Mobbing (Hm), and with the task carried out by the T9 (hole drilling) workers. Hence, this construction task is associated with a lack of Demands and Recognition in these workers, an excessive level of emotional involvement in performing the task, and the possibility of Mobbing. This again seems logical because the workers carrying out the hole-drilling task usually work alone, or at most with one other operator; after setting out the plot where the greenhouse is to be built, the operator only enters it to carry out the drilling and only interacts with the rest of the crews in a very specific way; therefore, if this contact involves no close relationship with or recognition from his peers, he may feel displaced (Mobbing), which can affect his health [94,95] and productivity [79].

Cluster 4 brings together those variable categories studied that have positive or low negative values in Dimension 2 with negative or low positive values in Dimension 1. It is characterised by associating a low level (L) of worker risk with the psychosocial factors Rhythm (Lr), Relationships (Lrl), Health (Ls), Recognition (Lrc), Autonomy (Lau), Support (Lap), Compensation (Lcp), Control (Lco), Mental Load (Lcm), Mobbing (Lm), and Emotional (Lem), and with smaller companies that have fewer workers (X2), workers of Eastern European nationality (N2), aged between 25 and 40 years old (E2) who have more than two children (H4) or no child (H1), and who have been carrying out greenhouse construction work for less than 5 years (Ñ1) or between 15 and 25 years (Ñ3), and working in the construction tasks T5 (masonry + foundations and casting + installation of wire elements + placement of guttering + placement of windows, assembly of multi-tunnel greenhouses + transport of materials and operators), T7 (installation of wire elements + placement of guttering), and T3 (masonry + foundations and casting + installation of wire elements + placement of guttering). This multiplicity of tasks that workers would perform who are associated with this cluster means they present a low level of risk in the psychosocial factors indicated, i.e., Rhythm, Mobbing, Relationships, Health, Recognition, Autonomy, Support, Emotional, Compensation, Control, and Mental Load. It is interesting to note that, as occurs in this cluster, foreign workers are recognised for their work (a low-risk level in the Recognition factor) and are not limited in their Autonomy (a low-risk level in the Autonomy factor). As Gyekye [97] points out, recognition is positively associated with worker satisfaction, which, in turn, affects their productivity.

### 5.5. Study Limitations

To characterise the psychosocial risk prevalence in workers in the greenhouse construction industry in south-eastern Spain, we considered a sample of 9.06% of the workers registered in Almería, with an accuracy of 5.72%. Therefore, the results could be modified if different workers had participated in the sampling. Consequently, the results set out here are an estimate. Another limitation of the study, related to the previous one, is that the sampling phase uses a self-reported questionnaire that is completed by the workers themselves.

Finally, a further study limitation is that when selecting the workers, they were not asked about their prior history of psychiatric or psychological disorders, which could have been grounds for their exclusion.

### 5.6. Proposed Recommendations to Prevent Psychosocial Risk Affection in Workers

As noted in Section 4.1.3., the average percentage of workers at high risk for all 12 factors is 2.9%, while the medium risk is 42.2%, with the rest presenting a low risk. Therefore, 45.1% of this average of workers have a medium-high risk level that requires some kind of short- and medium-term intervention.

In this section, a number of recommendations are proposed, the objective of which is to avoid the emergence of medium-high psychosocial risk levels in workers in the Almerian greenhouse construction industry:

1. New prevention programmes or networks should be created that are capable of helping workers, especially immigrants, facilitating their family conciliation and adapting them to new cultures and living conditions, as indicated by Montoya-García [65]. This recommendation is mainly addressed to immigrant workers in Cluster 1, who present an average level of risk for various psychosocial factors, as well as those workers in Cluster 4. In these programmes, greenhouse construction employers should actively participate in order to learn how to value the work of their employees and thus promote their psychosocial well-being. The specific measures that could be part of these prevention programmes should include salary bonuses, an agreed adjustment to the working day (the start and end) that makes it possible to reconcile work and family life better, and improved resting places at work.
2. Organising training courses and activities in which employers and employees can interact outside of work [98]. Recommended for all workers in all clusters.
3. Avoiding the individual working alone full time and encouraging shifts in which at least two people are involved, alternating activities as they complete the task, with additional breaks provided during the working day [99]. A recommendation aimed primarily at workers in Clusters 2 and 3 since they perform tasks individually in most cases.
4. Providing training courses (and ergonomic training) for workers to prevent psychosocial risks. Recommended for workers in all clusters, but mainly those in which immigrants predominate (Clusters 1 and 4).
5. Reorganising the work when necessary. Recommended for all workers in all clusters.
6. Performing tasks with many workers, where possible, and over short periods of time. The cost of the work will be the same since the employer pays for the hours worked. Recommended for all workers in all clusters, although it would be preferable, as far as possible, for those in Clusters 2 and 3, who perform the tasks individually.

## 6. Conclusions

The average greenhouse construction company in the province of Almería is a small limited company, whose corporate regime is that of *Sociedad Limitada*, mainly assigned to the 4399 national economic activities code (other specialised construction activities, not elsewhere specified), which operates 12 months a year, with an annual turnover of less than EUR 2 million (69.3%), which mainly constructs Almería-type greenhouses, which has been carrying out its construction activity for an average of 21.8 years, with an average of 22.8 workers, and an average of 16.0 immigrant workers, predominantly Romanians, and that forms specialised crews to carry out the various construction and maintenance tasks.

The average age of greenhouse construction workers in the province of Almería is 39.84 years old, although 3.2% are under the age of 25 years old. Of these, 100% are men, with an average weight of 83.16 kg, an average height of 1.76 m, who are mainly married and, to a lesser extent, single, with an average of 1.06 children, predominantly Romanian workers and those from other Eastern European countries, who work in specialised crews carrying out specific construction tasks. Of these, 85.5% have not suffered any accidents during the last year, and 90.3% have not suffered any occupational illness in the last year as a result of greenhouse construction, and they have been building or maintaining greenhouses for an average of 13.27 years.

The assessment conducted using the MPF method shows that Almerian greenhouse construction workers have a medium-high risk level (45.1%) for the average percentage of the combined 12 factors assessed by this method, thus requiring some form of short- and medium-term intervention. Only 2.9% of workers are at high risk.

Of the 12 psychosocial factors assessed using the MPF method, 7 of them presented a high level of worker risk: Mobbing (3.2%), Relationships (1.6%), Recognition (1.6%), Autonomy (12.9%), Emotional (8.0%), Control (4.8%), and Demands (3.2%).

Through the multiple correspondence analysis technique, variables have been grouped into four clusters, correlating larger companies with a medium- (workers over 40 years of age or less than 25 years of age) to high-risk level (workers under 25 years of age) in several of the psychosocial factors assessed in Spanish workers, whereas in smaller companies, the workers are on average between 25 and 40 years old and Eastern European or African, presenting a low- or high-risk level, depending on the psychosocial factors and the tasks performed.

New prevention programmes are needed to optimise the psychosocial conditions of Almerian greenhouse construction workers, which involve the workers themselves, their employers, and other social actors.

Lastly, as a continuation to this research work, we propose a joint assessment of psychosocial and musculoskeletal risks in Almerian greenhouse construction workers, given the relationship between them, as highlighted by several authors [54,55,56].

## Figures and Tables

**Table 1:** Scales for evaluating the scores of each MPF-method variable [65,66].

MPF-Method Variables	Ratios
Rhythm, Mobbing, Relationships, Health, Recognition, Autonomy, Emotional involvement, Support, Compensation, Control	≥1 <4 = High risk (H) ≥4 but ≤7 = Medium risk (M) >7 but ≤10 = Low risk (L)
Demands (of work)	≤1 <4 = Low risk (L)
	≥4 but ≤7 = Medium risk (M)
	>7 but ≤10 = High risk (H)
Mental Load	<1 <7 = High risk (H)
	≥7 but ≤14 = Medium risk (M)
	>14 but ≤20 = Low risk (L)

Adapted with permission from Montoya-García et al. (2013). Copyright 2012 Elsevier Ltd. and The Ergonomics Society. Adapted with permission from Ruiz and Idoate (2005). Copyright 2005 Ruiz and Idoate.

**Table 2:** Descriptive parameters of company workers variables (average (s.d.) and frequency).

Variables/Variable Categories	Average (s.d.)/Frequency (%)
Worker’s age (years)	39.84 (±9.70)
<25	3.2
25–40	54.9
>40	41.9
Worker’s sex	
Male	100.0
Female	0.0
Worker’s weight (kg)	83.16 (±11.50)
W < 70	11.3
70 ≤ W < 80	30.6
80 ≤ W < 90	29.0
90 ≤ W < 100	22.6
W ≥ 100	6.5
Workers’ height (m)	1.76 (±0.06)
H < 1.7	9.7
1.7 ≤ H < 1.8	53.2
1.8 ≤ H < 1.9	37.1
Workers’ marital status	
Married	53.2
Single	33.9
Divorced	4.8
Common-law partner	8.1
Number of workers’ children	1.06 (±1.18)
0	43.5
1	24.2
2	19.4
>2	12.9
Nationality of workers	
Spanish	38.7
Eastern European ^1^	59.7
African ^2^	1.6
Specialisation of workers in greenhouse construction *	
8	6.5
7	8.1
3 + 4 + 5 + 6	14.5
4 + 11	4.8
3 + 4 + 5 + 6 + 9 + 10 + 11	8.1
9	1.6
5 + 6	43.5
4 + 5 + 6 + 7 + 9	9.7
2	3.2
Accidents suffered by workers during greenhouse construction in the last year	
No accident	85.5
Fall from height	1.6
Hit and fall	4.8
Contact with thermal agent	3.3
Fall to the same level	4.8
Accidents suffered by workers outside their jobs in the last year	
No accident	93.6
Traffic accident	1.6
Trip	3.2
Fall to the same level	1.6
Period off work for workers caused by accident in their workplace in the last year (days)	
0	95.2
1–7	1.6
8–30	3.2
Period off work for workers caused by accident outside their work in the last year (days)	
0	95.2
1–7	3.2
8–30	1.6
Workers’ illnesses caused by carrying out their work in greenhouse construction	
No	90.3
Lower back pain	8.1
Herniated disc	1.6
Years that workers have been constructing greenhouses	13.27 (±8.73)
<5	11.3
5–14	48.4
15–24	27.4
≥25	12.9

* The legend for each number’s meaning is shown in Table A3. Nationality: ^1^ Eastern European (Romanians and Russians), ^2^ African (Moroccans and Malians).

**Table 3:** Descriptive analysis of the scores obtained for the 12 psychosocial factors evaluated by MPF.

Psychosocial Factor	Minimum	Maximum	Average	Standard Deviation	Risk Level
Rhythm	4.00	10.00	7.52	1.66	L
Mobbing	3.00	10.00	8.56	1.65	L
Relationships	3.70	10.00	7.44	1.54	L
Health	5.00	10.00	7.60	1.72	L
Recognition	1.00	10.00	6.97	1.68	M
Autonomy	1.00	10.00	6.53	2.33	M
Emotional	1.00	10.00	7.02	2.51	L
Support	5.30	10.00	8.18	1.11	L
Compensation	4.00	9.00	7.08	1.15	L
Control	3.70	9.30	7.17	1.66	L
Demands	4.00	7.30	5.88	0.87	M
Mental load	9.20	19.80	16.56	2.87	L

**Table 4:** Frequency and mode for the different risk categories of the psychosocial factors evaluated by MPF for Almerian greenhouse construction workers.

Psychosocial Factor	Risk Category	Frequency (%)	Psychosocial Factor	Risk Category	Frequency (%)
**Rhythm**	H	0.0	**Emotional**	H	8.0
	M	45.2		M	45.2
	L *	54.8		L *	46.8
**Mobbing**	H	3.2	**Support**	H	0.0
	M	12.9		M	16.1
	L *	83.9		L *	83.9
**Relationships**	H	1.6	**Compensation**	H	0.0
	M	41.9		M	48.4
	L *	56.5		L *	51.6
**Health**	H	0.0	**Control**	H	4.8
	M	41.9		M	45.2
	L *	58.1		L *	50.0
**Recognition**	H	1.6	**Demands**	H	3.2
	M *	50.0		M *	96.8
	L	48.4		L	0.0
**Autonomy**	H	12.9	**Mental Load**	H	0.0
	M	37.1		M	25.8
	L *	50.0		L *	74.2

* Mode.

**Table 5:** Discrimination measures of the variables in the multiple correspondence analysis.

Variables	Dimension		Average
	1	2	
Worker’s age (E)	0.075	0.362	0.218
Worker’s marital status (C)	0.033	0.269	0.151
Number of children (H)	0.019	0.189	0.104
Nationality (N)	0.079	0.064	0.072
Worker activity (A)	0.372	0.482	0.427
No. of years worked (Ñ)	0.043	0.457	0.250
Rhythm (R)	0.446	0.034	0.240
Mobbing (M)	0.004	0.264	0.134
Relationships (Rl)	0.636	0.049	0.342
Health (S)	0.336	0.004	0.170
Recognition (Rc)	0.639	0.025	0.332
Autonomy (Au)	0.321	0.118	0.220
Emotional (Em)	0.535	0.013	0.274
Support (Ap)	0.069	0.277	0.173
Compensation (Cp)	0.573	0.000	0.287
Control (Co)	0.595	0.061	0.328
Demands (De)	0.067	0.013	0.040
Mental Load (CM)	0.336	0.359	0.348
Company activity (Z)	0.184	0.171	0.178
No. of company workers (X)	0.353	0.342	0.348
No. of immigrant workers in company (Y)	0.295	0.330	0.312
Company turnover (F)	0.482	0.262	0.372
Active Total	6.490	4.146	5.318
% of Variance	29.502	18.846	24.174

**Table 6:** Description of the four identified clusters of the variable categories.

Variables	Cluster 1	Cluster 2	Cluster 3	Cluster 4
Worker’s age (E)	<25 or >40	<25		25–40
Worker’s marital status (C)	-	Single	Common-law partner	Married or Divorced
Number of children (H)	1	-	-	0 or 2 or >2
Nationality (N)	Spanish	-	-	Eastern European
Worker activity (A)	T2 (workers who put on and change greenhouse plastic) and T8 (foundations and casting + installation of wire elements + placement of gutters + changing of plastic + placement of windows)	T4 (Foundations and casting)	T9 (Window placement)	T5 (masonry + foundations and casting + installation of wire elements + placement of guttering + placement of windows, assembly of multi-tunnel greenhouses + transport of materials and operators), T7 (installation of wire elements + placement of guttering), and T3 (masonry + foundations and casting + installation of wire elements + placement of guttering)
No. of years worked (Ñ)	≥25	-	-	<25
Company activity (Z)	All kinds of greenhouses and other activities	-	-	Almería-type greenhouse
No. of company workers (X)	>50	-	-	10–19
No. of immigrant workers in company (Y)	20–50	-	-	<20
Company turnover (mill. €) (F)	≥5	-	-	<2
Rhythm (R)	Medium	-	-	Low
Mobbing (M)		-	High	Low
Relationships (Rl)	Medium	High	-	Low
Health (S)	Medium	-	-	Low
Recognition (Rc)	Medium	-	High	Low
Autonomy (Au)	Medium	High		Low
Emotional (Em)	Medium	-	High	Low
Support (Ap)	-	Medium	-	Low
Compensation (Cp)	Medium	-	-	Low
Control (Co)	Medium	High	-	Low
Demands (De)	-		High	-
Mental Load (CM)	Medium	Medium	-	Low

## Appendix A

**Table A1:** Decision matrix for selecting the assessment methods.

Method	Rapidity in Filling out the Questionnaire	Number of Questions in the Questionnaire	Applicability to the Construction Sector	Reliability and Ease of Statistical Analysis	Score Total
FPSICO [67]	2	3	3	4	12
ISTAS21 [68]	2	3	3	3	11
MPF [66]	4	4	3	2	13
FP-ISR [69]	3	3	2	3	11
PSICOMAP [70]	2	3	2	3	10
RED-WONT [71]	2	2	4	4	12

**Table A2:** Frequency of the company variable categories.

Variables/Variable Categories	Frequency (%)
Company corporate regime	
Limited Company	100.0
Public Limited Company	0.0
Other types	0.0
Type of construction activity	
Only constructing Almería-type greenhouses	46.1
Building all kinds of greenhouses	15.4
Building greenhouses and other activities	38.5
Age of the construction company (years)	
0–9	7.7
10–19	38.4
20–30	30.8
>30	23.1
Total number of workers	
<10	15.4
10–19	61.5
20–50	7.7
>50	15.4
Total number of immigrant workers	
<10	38.5
10–19	46.1
20–50	7.7
>50	7.7
Nationality of immigrant workers	
Romanians	46.1
Romanians and sub-Saharans ^1^	7.7
Romanians, Russians, and North Africans ^2^	38.5
Romanians, Russians, North Africans, and Latin Americans ^3^	7.7
Number of office workers	
0	23.1
1–5	69.2
6–10	7.7
Number of workers on-site	
<10	23.1
10–19	61.5
20–50	0.0
>50	15.4
Crews of workers are formed that carry out specialised work	
Yes	100.0
No	0.0
Type of crews the company has *	
3 + 4 + 5 + 8 + 11	15.4
3 + 4 + 5 + 6 + 8 + 9 + 11	15.4
3 + 4 + 5 + 6 + 7 + 8 + 9 + 10 + 11	7.7
3 + 4 + 5 + 6 + 8 + 9 + 10 + 11	15.4
6 + 9 + 10 + 11	7.7
2 + 3 + 4 + 5 + 6 + 7 + 8 + 9 + 11	15.4
2 + 3 + 4 + 5 + 6 + 7 + 8 + 9 + 10 + 11	23.0
Annual period of activity of the company	
<1 Year	0.0
Annual	100.0
Annual company turnover (millions)	
0.5 < F	0.0
0.5 ≤ F < 1.0	46.2
1.0 ≤ F < 2.0	23.0
2.0 ≤ F < 3.0	0.0
3.0 ≤ F < 5.0	7.7
F ≥ 5.0	23.1
Code of Economic Activity Assigned (CNAE)	
4121	7.7
4391	38.5
4399	53.8

* The legend for each number’s meaning is shown in Table A3. Nationality: ^1^ sub-Saharans (mainly Malians), ^2^ North Africans (mainly Moroccans), ^3^ Latin Americans (mainly Argentines).

**Table A3:** Construction tasks of the companies and their nomenclature.

Construction Task	Nomenclature Codes Table 2 and Table A4
Ground levelling	1
Hole drilling	2
Masonry	3
Foundations and casting	4
Wire Elements (Wire Structure)	5
Placing rain guttering	6
Plastic	7
Welding of metallic elements	8
Window placement	9
Constructing multi-tunnel greenhouses	10
Transport of materials and operators	11

**Table A4:** Nomenclature for the general company categories and the worker variable categories used in the multiple correspondence analysis.

Variable-Category	Nomenclature	Variable-Category	Nomenclature
**Worker’s age**	**E**	**No. of years worked**	**Ñ**
<25	E1	Ñ < 5	Ñ1
25–40	E2	5 ≤ Ñ < 15	Ñ2
>40	E3	15 ≤ Ñ < 25	Ñ3
		≥25	Ñ4
**Worker’s marital status**	**C**	**Company activity**	**Z**
Married	C1	Industrial type greenhouse	Z1
Single	C2	Almería-type greenhouse	Z2
Divorced	C3	Industrial greenhouse + Almería type	Z3
Common-law partner	C4	All kinds of greenhouses and other activities	Z4
Widower	C5		
**Number of children**	**H**	**No. of company workers**	**X**
0	H1	<10	X1
1	H2	10–19	X2
2	H3	20–50	X3
>2	H4	>50	X4
**Nationality**	**N**	**No. of immigrant workers in company**	**Y**
Spanish	N1	<10	Y1
Eastern European	N2	10–19	Y2
African	N3	20–50	Y3
Latin American	N4	>50	Y4
**Worker activity ***	**A**	**Company turnover (mill. €)**	**F**
8	A1	0.5 ≤ F < 1.0	F1
7	A2	1.0 ≤ F < 2.0	F2
3 + 4 + 5 + 6	A3	F ≥ 5.0	F3
4 + 11	A4		
3 + 4 + 5 + 6 + 9 + 10 + 11	A5		
9	A6		
5 + 6	A7		
4 + 5 + 6 + 7 + 9	A8		
2	A9		

* Nomenclature of the worker’s work activity, as described in Table A3.

**Table A5:** Nomenclature of the risk level categories for each of the 12 assessed psychosocial factors used in the multiple correspondence analysis.

Variable-Category	Nomenclature	Variable-Category	Nomenclature
**Rhythm**	**R**	**Emotional**	**Em**
High	Hr	High	Hem
Medium	Mr	Medium	Mem
Low	Lr	Low	Lem
**Mobbing**	**M**	**Support**	**Ap**
High	Hm	High	Hap
Medium	Mm	Medium	Map
Low	Lm	Low	Lap
**Relationships**	**Rl**	**Compensation**	**Cp**
High	Hrl	High	Hcp
Medium	Mrl	Medium	Mcp
Low	Lrl	Low	Lcp
**Health**	**S**	**Control**	**Co**
High	Hs	High	Hco
Medium	Ms	Medium	Mco
Low	Ls	Low	Lco
**Recognition**	**Rc**	**Demands**	**De**
High	Hrc	High	Hde
Medium	Mrc	Medium	Mde
Low	Lrc	Low	Lde
**Autonomy**	**Au**	**Mental Load**	**CM**
High	Hau	High	Hcm
Medium	Mau	Medium	Mcm
Low	Lau	Low	Lcm

## Appendix B

**Questionnaire**

**I. General Company Characteristics (For employers)**

(The questionnaire is anonymous)

P.0. Municipality: .....................................

P.1. Corporate regime:

  □ Limited Company

  □ Public Limited Company

  □ Others (indicate type): ........................................

P.2. Type of activity carried out by the company:

  □ Constructing only industrial greenhouses

  □ Constructing only Almería-type greenhouses

  □ Constructing both types of greenhouses

  □ Constructing greenhouses and other activities (irrigation, air-conditioning, etc.): ..............................

P.3. Number of years constructing greenhouses: .....................

P.4. Total number of workers in the company: .....................

P.5. Total number of immigrant workers in the company: .......................

P.6. Specify the nationality of immigrant workers and their number:

  □ African..................

  □ Eastern European..................

  □ Latin American..............

  □ Others (specify) .........................

P.7. Number of office or administration workers: .....................

P.8. Number of on-site workers: ....................

P.9. Are there specialised work crews:

  □ No

  □ Yes

P.10. If worker crews are formed, indicate which (or indicate if they are subcontracted):

  □ Ground levelling

  □ Hole drilling

  □ Masonry

  □ Foundations and casting

  □ Wire Elements (Wire Structure)

  □ Placing rain guttering

  □ Plastic

  □ Welding metallic elements

  □ Window placement

  □ Transport of materials and operators

  □ Others (specify) .........................

P.11. No. of months of activity throughout the year: ......................

P.12. Annual company turnover, in euros (F):

  □ < 0.5 million €

  □ 0.5 < F < 1.0 million €

  □ 1.0 < F < 2.0 million €

  □ 2.0 < F < 3.0 million €

  □ 3.0 < F < 5.0 million €

  □ F > 5.0 million €

P.13. Indicate the National Classification of Economic Activities (CNAE): ............................

**II. General Characteristics of the Worker (For workers)**

(The questionnaire is anonymous)

P.0. Age: .....................................

P.1. Sex:

  □ Male

  □ Female

P.2. Weight: ..............................

P.3. Height: ...........................

P.4. Marital status:

  □ Married

  □ Single

  □ Divorced

  □ Common-law partner

  □ Widower

P.5. Number of children in your care: ......................

P.6. Nationality of the worker: .......................

P.7. Which greenhouse construction work activity do you carry out?

  □ Ground levelling

  □ Hole drilling

  □ Masonry

  □ Foundations and casting

  □ Wire Elements (Wire Structure)

  □ Placing rain guttering

  □ Plastic

  □ Welding metallic elements

  □ Window placement

  □ Transport of materials and operators

  □ Others (specify) .........................

P.8. Have you suffered an accident at work in the last year?

  □ No

  □ Yes (Indicate accident type): .........................

P.9. Have you suffered an accident at home or in other activities outside work in the last year?

  □ No

  □ Yes (Indicate accident type): .........................

P.10. Have you been off work as the result of an accident at work in the last year?

  □ No

  □ Yes (Indicate the period off work in number of days or months, as appropriate): .........................

P.11. Have you been off work as a result of an accident outside of work in the last year?

  □ No

  □ Yes (Indicate the period off work in number of days or months, as appropriate): .........................

P.12. Have you suffered any illness as a result of your work constructing greenhouses?

  □ No

  □ Yes (Indicate the illness): .........................

P.13. How many years have you been working in greenhouse construction? .....................

**III. MPF Questionnaire for Psychosocial Risks at Work (For workers)**

(The questionnaire is anonymous)

**1. Is your health satisfactory?**

1-not at all-/very little-2   3-a little-4   5-normal-6   7-quite a lot-8   9-a lot-10

**2. Are your relationships with co-workers good in general?**

1-not at all-/very little-2   3-a little-4   5-normal-6   7-quite a lot-8   9-a lot-10

**3. Is your work pleasant?**

1-not at all-/very little-2   3-a little-4   5-normal-6   7-quite a lot-8   9-a lot-10

**4. Do you have enough time to perform your tasks?**

1-not at all-/very little-2   3-a little-4   5-normal-6   7-quite a lot-8   9-a lot-10

**5. Can you decide on some aspects of your work tasks? **

1-not at all-/very little-2   3-a little-4   5-normal-6   7-quite a lot-8   9-a lot-10

**6. Are there tensions at work because of other teammates?**

1-not at all-/very little-2   3-a little-4   5-normal-6   7-quite a lot-8   9-a lot-10

**7. Do you usually have breaks during your work?**

1-not at all-/very little-2   3-a little-4   5-normal-6   7-quite a lot-8   9-a lot-10

**8. Is your effort at work recognised by your superiors?**

1-not at all-/very little-2   3-a little-4   5-normal-6   7-quite a lot-8   9-a lot-10

**9. Do you have sufficient means to perform your task?**

1-not at all-/very little-2   3-a little-4   5-normal-6   7-quite a lot-8   9-a lot-10

**10. Can you concentrate on your work?**

1-not at all-/very little-2   3-a little-4   5-normal-6   7-quite a lot-8   9-a lot-10

**11. Are you over-emotionally involved in your work?**

1-not at all-/very little-2   3-a little-4   5-normal-6   7-quite a lot-8   9-a lot-10

**12. Can you do the tasks at an appropriate pace?**

1-not at all-/very little-2   3-a little-4   5-normal-6   7-quite a lot-8   9-a lot-10

**13. Does anyone on the team repeatedly treat any of your teammates badly?**

1-not at all-/very little-2   3-a little-4   5-normal-6   7-quite a lot-8   9-a lot-10

**14. Are you overloaded by the amount of work you do?**

1-not at all-/very little-2   3-a little-4   5-normal-6   7-quite a lot-8   9-a lot-10

**15. Do you have the means to propose improvements to your work?**

1-not at all-/very little-2   3-a little-4   5-normal-6   7-quite a lot-8   9-a lot-10
